# Couples’ psychological adjustment to twin parenthood: mode of conception (spontaneous versus assisted reproduction) and gender differences

**DOI:** 10.1017/S1463423618000269

**Published:** 2018-04-30

**Authors:** Iva Tendais, Bárbara Figueiredo, Catarina Canário, David A. Kenny

**Affiliations:** 1 School of Psychology, Department of Applied Psychology, University of Minho, Braga, Portugal; 2 Department of Psychology, University of Connecticut, Storrs, CT, USA

**Keywords:** anxiety, assisted reproduction, depression, infertility, marital relationship

## Abstract

**Aim:**

To examine whether mode of conception and gender are associated with parents’ psychological adjustment across the transition to twin parenthood.

**Background:**

There is limited knowledge on the psychological adjustment of couples to twin parenthood during pregnancy and early *postpartum*, especially for fathers. The available research suggests that first-time mothers of twins conceived by assisted reproduction techniques (ART) may experience lower psychosocial well-being than mothers of spontaneously conceived (SC) twins.

**Methods:**

A total of 41 couples expecting twins, 25 of whom conceived spontaneously and 16 conceived by assisted reproduction techniques, completed measures of depressive and anxiety symptoms, marital relationship, attitudes to sex, and attitudes to pregnancy and the baby.

**Findings:**

ART parents showed a decline in marital relationship quality, no changes in attitudes to pregnancy and the baby and no changes in attitudes to sex over the *postpartum*. In contrast, SC parents did not change their perception of the marital relationship, reported more positive attitudes to pregnancy and the baby, and more positive attitudes to sex over the *postpartum*. Compared with the other groups (SC mothers and fathers, ART fathers), ART mothers exhibited a higher increase in depressive and anxiety symptoms from pregnancy to *postpartum* and only anxiety symptoms exhibited a decline trend over the *postpartum*. These findings suggest that ART parents may experience more psychological difficulties during the transition to twin parenthood than SC parents. ART mothers, in particular, appear to be more at risk of high levels of *postpartum* depressive symptoms.

## Introduction

Twin birth rates have increased worldwide in the last decades, largely due to the increased use of assisted reproduction technology (ART). Parents of twins have to deal repeatedly with several stressful situations. Twins have higher risk of prematurity, low birth weight, and perinatal mortality compared with singletons (Blondel *et al*., 2002). Twins also show poorer neurodevelopmental outcomes than singletons (Lorenz, 2012). To meet the needs of two or more children, parents may experience financial, childcare, and physical and psychological issues (Ellison and Hall, 2003; Choi *et al*., 2009). The first three months after delivery are a particularly vulnerable period for mothers due to the overload of caregiving tasks and different sleeping and feeding patterns of the twins (Beck, 2002).

Given that infertility-related distress has been observed during pregnancy and *postpartum* among parents of ART singletons (Hjelmstedt *et al*., 2004), it would be expected that twin parenthood after ART would have a cumulative negative effect on parents’ psychological adjustment. However, some studies suggest that ART parents of twins have similar or even fewer psychological symptoms than parents of spontaneously conceived (SC) twins (Munro *et al*., 1990; Vilska *et al*., 2009). One of the few longitudinal studies that examined the impact of mode of conception on the psychological adjustment of twins’ parents found that ART mothers exhibited fewer depressive symptoms at mid-pregnancy and similar levels of depressive and anxiety symptoms compared with SC mothers at three months and one year *postpartum*; however, no differences were found between ART and SC fathers of twins at pregnancy or *postpartum* (Vilska *et al*., 2009). Other studies revealed that first-time mothers of ART twins reported more psychological symptoms and poorer coping resources than first-time mothers of SC twins (Colpin *et al*., 1999; Baor *et al*., 2004; Baor and Soskolne, 2010). Although some studies found that primiparous ART women had more psychological symptoms and lower psychosocial well-being than multiparous ART mothers (Colpin *et al*., 1999; Baor *et al*., 2004; Baor and Soskolne, 2010), others found no evidence for negative effects of parity on parental mental health (Vilska *et al*., 2009).

With regard to the marital relationship, the few studies that have compared ART and SC parents of twins have found lower self-reported marital quality in ART mothers at late pregnancy compared with mothers of SC twins (Baor and Soskolne, 2010), but similar marital relationship quality nine months (Baor *et al*., 2004) and one year *postpartum* (Colpin *et al*., 1999).

Differences in pregnancy and child-related attitudes between parents of ART and SC twins are largely unknown. Research with singletons suggests that ART mothers seem to have more idealized attitudes to pregnancy than SC mothers (McMahon *et al*., 1999). It is unclear whether the prenatal maternal expectations of ART mothers of twins are unrealistic as well (Baor and Soskolne, 2010).

There is also a very limited body of research on gender differences in psychological adjustment to twin parenthood among ART and SC parents. Munro *et al*. (1990) found no gender differences in psychiatric morbidity in a sample of parents of twins conceived spontaneously, by hormonal treatment or IVF. In contrast, mothers of twins showed lower psychological well-being than fathers, regardless of mode of conception (Baor *et al*., 2004).

The inconclusive evidence regarding parents’ psychological adjustment to twin parenthood after ART may be at least in part explained by differences in studied samples. Some studies have included only primiparous women (McMahon *et al*., 1999; Baor and Soskolne, 2010), whereas in others multiparous women were also included (Munro *et al*., 1990; Colpin *et al*., 1999; Baor *et al*., 2004; Vilska *et al*., 2009). ART couples appear to be a heterogeneous group in terms of their socio-demographic characteristics and fertility difficulties (Hammarberg *et al*., 2008).

The purpose of this study was to examine whether mode of conception and gender are associated with parents’ psychological adjustment (anxiety and depressive symptoms, marital relationship quality, attitudes to pregnancy and the baby and attitudes to sex) across the transition to twin parenthood, taking into account the interdependence of data within couples. Based on previous research, we hypothesized that ART parents (and ART mothers in particular) would have poorer psychological adjustment than SC parents during the transition to twin parenthood.

## Method

### Procedures and participants

After receiving ethical approval from the Ethics Committee, consecutive couples expecting twins were recruited at the antenatal Obstetrical Units of three public hospitals in Northern Portugal. Inclusion criteria included twin pregnancy, less than 15 weeks gestational age and knowing how to read and write in Portuguese. Participants provided written informed consent. Information on couples’ psychological adjustment was collected three times during pregnancy (T1–T3) and three times after childbirth (T4–T6). The measures were completed separately by women and men, on average, at 13, 21 and 30 weeks of pregnancy and 1, 4 and 8 weeks after birth.

The sample included 41 couples, 25 of whom with twins conceived spontaneously (SC) and 16 conceived by assisted reproduction techniques (ART). Of the 50 eligible couples approached, 45 agreed to participate in the study (80%). Four couples were excluded due to death of one or both twins. Two couples dropped out the study after childbirth for personal reasons. Within the ART group, seven couples conceived through *in vitro* fertilization, eight by intracytoplasmic sperm injection and one by ovarian stimulation medication.

### Measures

Couples’ information was collected using a socio-demographic questionnaire.

The state subscale of the State-Trait Anxiety Inventory Form Y (STAI; Spielberger *et al*., 1983) was used to measure anxiety symptoms. It comprises 20 items that are scored on a four-point Likert scale. The scores range from 20 to 80, with higher scores indicating higher anxiety. The Portuguese version of the STAI has been validated for use during pregnancy and the *postpartum* period (Tendais *et al*, 2014). In this study, the Cronbach’s *α* was 0.93 for both men and women.

The Edinburgh Postnatal Depression Scale (EPDS; Cox *et al*., 1987) was used to measure depressive symptoms. The EPDS is a self-report questionnaire composed of 10 items scored on a four-point Likert scale addressing depressive symptoms within the previous seven days. The scores range from zero to 30, with higher scores indicating more severe depressive symptoms. The Portuguese version of the EPDS has been validated both for pregnancy and the *postpartum* period (Tendais *et al*, 2014). In this study, the Cronbach’s *α* was 0.82 for women and 0.76 for men.

Marital relationship, attitudes to sex and to pregnancy and the baby were assessed with the subscales of the maternal and the paternal attitudes and adjustment scale (MAMA, PAPA) (Kumar *et al*., 1984). The attitudes to sex subscale comprises 12 items (range 12–48). The marital relationship and the attitudes to pregnancy and the baby subscales comprise 11 items each (range 11–44). Sample questions include ‘Has there been tension between you and your partner?’ (marital relationship), ‘Have you found your partner sexually desirable?’ (attitudes to sex), ‘Have you been worrying that you might not be a good mother/father?’ (attitudes to pregnancy and the baby). Each item is scored on a four-point Likert scale (1=never to 4=very often). The Portuguese version of the MAMA (Figueiredo *et al*., 2004) and PAPA (Pinto *et al*., 2017) have been validated both for pregnancy and the *postpartum*. In this study, the Cronbach’s *α* was 0.84 for MAMA and 0.81 for PAPA.

All of these measures have been used in previous studies with ART and SC parents (Pinto *et al*., 2017; 2018; Tendais and Figueiredo, 2016).

### Data analysis strategy

Pearson’s correlations were used to assess couples’ interdependence at baseline. In addition, unconditional multilevel models with gender and time as fixed effects were run for all outcome measures to estimate couples’ interdependence over time.

Dyadic linear growth curve models were estimated using multilevel modeling (Kenny *et al*., 2006), a method for studying hierarchically nested data structure such as repeated measures from dyads. Growth curve models were used to evaluate the degree of change in an outcome variable using time as the predictor variable and the influence of other variables in the moderation of change over time (Kashy *et al*., 2008). The data consist of 492 potential observations: 41 couples by six time points by two people (husband and wife). Two-level models were estimated for each psychological adjustment variable (dependent variables) in which observation is level one and couple is level two.

Based on the examination of mean scores over time for the sample for depression, anxiety and attitudes to sex, there was a large change at the first assessment *postpartum*. Thus, for these variables, we used piecewise models with two different slopes: the first, from three (T1) to nine months (T4) and the second, from nine months to the last assessment (T6). In addition, the model contains two intercepts. The first ‘intercept’ represented where a person started at T1, and a second intercept named ‘pre-postnatal transition’ reflects change from pregnancy to the *postpartum* period. For the remaining outcome variables (marital relationship and attitudes to pregnancy and the baby), a single linear trend was sufficient to model changes from the first assessment during pregnancy to the last assessment *postpartum*. The models included main effects for time, mode of conception and gender. All interactions were examined, resulting in a possible three-way interaction between time, mode of conception and gender. Three multivariate outliers for anxiety and two for depression were detected and excluded from the analysis.

The time variable was defined in months and Time 0 was the beginning of the study (T1, *M*=3.05, SD=0.17) and so the intercept or baseline score represents the estimated mean level of each outcome variable at three months of pregnancy and the slope corresponds to the average linear change for each one month.

## Results

In the ART group, couples had been infertile for 3.8 years (SD=2.2) and received an average of 1.8 (SD=0.9) fertility treatments. On average, participants were 30.7 years old (SD=4.7, range 20–43 years), had been in the relationship for 8.3 years (SD=4.7, range 1–21 years), and the majority were Caucasian (97.6%), primiparous (78%), married (75.6%), employed (84.5%), did not complete high school or equivalent (51.2%), and had medium and medium-low socioeconomic level (29.3% and 41.5%, respectively). More than half of the women had complications during pregnancy (53.7%) and delivered by cesarean section (63.4%). The mean gestational age was 36.1 weeks (SD=2.7, range 27.7–38.7).

Most of the twins were born full-term (51.2%) and had low and very low birth weight (50% and 11%, respectively). In more than a third of cases, at least one of the twins was admitted to a neonatal intensive care unit (NICU) (34.1%) with an average stay of 21.3 days (SD=24.9).

ART twins had a significantly longer stay at NICU than SC twins (*U*=102.0, *P*=0.02). A significant association was found between mode of conception and parity (*χ* ^2^(1)=–7.38, *P*<0.01). All women in the ART group were primiparous, whereas in the SC group more than a third was multiparous (36.0%). No statistically significant differences were found for age, education, socioeconomic level, relationship length, pregnancy complications, delivery mode, gestational age or NICU admittance between SC and ART groups (32% versus 37.5%, respectively).

Means and standard deviations for the outcome variables are presented for women and men and for spontaneous and ART groups in Table 1.

**Table 1:** Mean and standard deviation of outcome variables for women and men and for spontaneous and assisted reproduction technique (ART) groups at all time points

	Women (*n*=41)	Men (*n*=41)	Spontaneous (*n*=50)	ART (*n*=32)
	*M* (SD)	*M* (SD)	*M* (SD)	*M* (SD)
STAI-T	37.2 (7.2)	32.4 (6.6)	34.5 (6.6)	35.3 (8.3)
STAI-S				
T1	37.4 (10.3)	34.6 (8.3)	35.6 (10.2)	36.7 (8.3)
T2	34.0 (8.0)	32.0 (9.1)	32.4 (8.9)	34.1 (8.0)
T3	35.9 (9.4)	33.6 (9.4)	34.0 (9.0)	36.1 (10.1)
T4	40.6 (12.7)	35.5 (12.4)	35.1 (12.1)	42.9 (12.4)
T5	36.4 (11.4)	34.0 (10.7)	33.0 (8.5)	40.4 (14.1)
T6	37.8 (13.5)	32.1 (8.4)	33.8 (10.9)	36.9 (12.6)
EPDS				
T1	6.3 (3.7)	4.6 (2.9)	5.8 (3.4)	5.0 (3.4)
T2	5.1 (3.0)	4.1 (3.1)	4.2 (3.0)	4.8 (2.9)
T3	5.8 (3.2)	4.6 (3.5)	4.8 (3.2)	5.3 (3.4)
T4	6.7 (5.2)	4.6 (3.5)	4.3 (3.6)	7.9 (5.0)
T5	6.1 (4.7)	4.0 (4.2)	4.3 (3.7)	7.1 (5.5)
T6	6.2 (4.5)	3.6 (3.2)	4.0 (3.5)	6.5 (4.6)
Attitudes to sex				
T1	36.6 (4.3)	39.0 (3.8)	37.9 (3.6)	37.7 (5.1)
T2	36.2 (4.2)	37.7 (4.3)	37.0 (3.9)	36.9 (4.8)
T3	35.7 (4.2)	36.9 (4.6)	36.9 (4.0)	35.4 (5.1)
T4	36.8 (4.9)	37.4 (4.6)	36.7 (5.1)	37.8 (4.0)
T5	36.8 (4.6)	40.5 (3.1)	39.5 (4.0)	36.3 (4.6)
T6	37.8 (4.9)	39.6 (3.5)	38.7 (4.7)	38.7 (4.0)
Attitudes to pregnancy and the baby				
T1	32.4 (2.8)	33.7 (3.9)	33.1 (3.6)	32.8 (3.2)
T2	33.9 (3.4)	34.1 (4.0)	34.2 (3.8)	33.7 (3.7)
T3	33.3 (2.9)	34.3 (3.5)	34.0 (2.8)	33.5 (3.8)
T4	34.3 (2.5)	34.5 (3.0)	34.6 (2.5)	34.1 (3.2)
T5	34.3 (3.5)	35.0 (3.3)	34.9 (2.4)	33.7 (5.1)
T6	34.6 (3.2)	35.2 (3.0)	35.2 (2.7)	34.3 (3.6)
Marital relationship				
T1	38.8 (4.4)	37.4 (3.5)	37.8 (3.9)	38.4 (4.1)
T2	38.4 (4.2)	37.1 (3.6)	37.6 (3.6)	37.9 (4.4)
T3	38.5 (4.2)	36.4 (3.6)	37.8 (3.9)	37.0 (4.3)
T4	38.3 (4.2)	37.2 (4.5)	38.1 (3.8)	37.1 (5.3)
T5	36.4 (5.9)	37.8 (4.5)	37.6 (4.9)	36.0 (6.3)
T6	37.0 (4.6)	37.0 (4.0)	36.9 (4.5)	37.2 (4.2)

STAI-T=State-Trait Anxiety Inventory – Trait; STAI-S=State-Trait Anxiety Inventory – State; EPDS=Edinburgh Postnatal Depression Scale; M=mean; SD=standard deviation. Data were collected at 13 (T1), 21 (T2) and 30 (T3) weeks of pregnancy and one (T4), four (T5) and eight (T6) weeks after birth.

Positive and significant correlations within couples were found at baseline and over time indicating within-couple similarity in psychological adaptation, that is, interdependence. At baseline, Pearson’s correlation between couples’ scores were significant for anxiety (*r*=0.29, *P*=0.01), depression (*r*=0.46, *P*<0.001), marital relationship (*r*=0.26, *P*=0.03), attitudes to sex, (*r*=0.31, *P*=0.01), and marginally significant for attitudes to pregnancy and the baby (*r*=0.23, *P*=0.49). Time-specific correlations of the residuals indicated that couples’ anxiety (*r*=0.28, *P*<0.001), depression (*r*=0.23, *P*<0.01), marital relationship (*r*=0.18, *P*=0.02), attitudes to sex (*r*=0.28, *P*<0.001), and attitudes to pregnancy and the baby (*r*=0.24, *P*=0.01), scores were significantly correlated over time.

Tables 2 and 3 include detailed information on estimates, standard errors and effect sizes of variables for the piecewise growth curve models adjusted for twins’ length of stay at NICU and mothers’ parity. The statistical information of additional analyses and random effects is presented in the text.

**Table 2:** Piecewise growth models for depressive and anxiety symptoms, attitudes to pregnancy and the baby and attitudes to sex from pregnancy to *postpartum*

	Anxiety			Depression			Attitudes to sex		
Fixed effects	*b*	SE	Effect size *r*	*b*	SE	Effect size *r*	*b*	SE	Effect size *r*
Intercept	32.77***	1.38		4.89***	0.53		37.99***	0.73	
Time pregnancy	−0.09	0.23	0.03	−0.14^†^	0.08	0.16	−0.28*	0.11	0.22
Pre-postnatal transition	3.90*	1.72	0.24	1.41*	0.56	0.25	1.43*	0.66	0.19
Time *postpartum*	−1.41*	0.62	0.19	−0.24	0.22	0.10	0.81**	0.28	0.24
Gender	−1.25	0.82	0.17	−0.72**	0.27	0.30	1.18**	0.38	0.37
Conception mode	0.97	1.18	0.11	−0.12	0.45	0.04	−0.06	0.61	0.01
Gender×conception mode	0.47	0.82	0.07	0.15	0.27	0.07	0.35	0.38	0.12
Time pregnancy×gender	0.14	0.22	0.06	−0.04	0.07	0.05	−0.11	0.09	0.10
Pre-postnatal transition×gender	−1.72	1.27	0.11	−0.36	0.48	0.08	0.39	0.53	0.06
Time *postpartum*×gender	0.14	0.60	0.02	−0.26	0.19	0.12	0.35	0.24	0.13
Time pregnancy×conception mode	−0.09	0.23	0.03	0.14^†^	0.08	0.15	−0.03	0.11	0.03
Pre-postnatal transition×conception mode	1.89	1.72	0.12	0.55	0.56	0.10	0.43	0.66	0.06
Time *postpartum*×conception mode	−0.20	0.62	0.03	−0.07	0.22	0.03	−0.55^†^	0.28	0.17
Time pregnancy×gender×mode	0.24	0.22	0.10	0.01	0.07	0.02	0.003	0.09	0.00
Pre-postnatal transition×gender×mode	−3.06*	1.27	0.19	−1.04*	0.48	0.21	−0.03	0.53	0.00
Time *postpartum*×gender×mode	1.24*	0.60	0.18	0.20	0.19	0.10	0.26	0.24	0.10
Random effects									
Variances	Women		Men	Women		Men	Women		Men
Intercept	41.77**		47.84***	7.47***		5.10***	11.55***		13.46***
Slope	71.45**		42.94**	12.14***		3.55**	7.07*		2.29
Residual	40.55***		24.38***	4.22***		2.84***	6.82***		5.72***

Gender was coded as men=1, women=−1; conception mode was coded as ART=1, spontaneous=−1; effect size *r*=√(*t* ^2^/[*t* ^2^+df]). Models are adjusted for twins’ length of stay at NICU and mothers’ parity. ^†^ *P*<0.10, *P<0.05, **P<0.01, ***P<0.001.

**Table 3:** Growth models for attitudes to pregnancy and the baby and marital relationship from pregnancy to *postpartum*

	Attitudes to pregnancy and the baby			Marital relationship		
Fixed effects	*b*	SE	Effect size *r*	*b*	SE	Effect size *r*
Intercept	33.14***	0.57		38.37***	0.67	
Time	0.17**	0.05	0.45	−0.19*	0.07	0.38
Gender	0.41	0.33	0.19	−0.88*	0.36	0.36
Conception mode	0.14	0.50	0.04	0.09	0.56	0.03
Gender×conception mode	−0.04	0.33	0.02	0.02	0.36	0.01
Time×gender	−0.03	0.04	0.11	0.11^†^	0.06	0.27
Time×conception mode	−0.10^†^	0.05	0.29	−0.15^†^	0.07	0.30
Time×gender×conception mode	0.01	0.04	0.04	0.04	0.06	0.11
Random effects						
Variances	Women		Men	Women		Men
Intercept	6.62**		15.19***	15.89***		8.15**
Slope	0.08*		0.13**	0.28**		0.10^†^
Residual	3.59***		2.53***	4.90***		5.78***

Gender was coded as men=1, women=−1; conception mode was coded as ART=1, spontaneous=−1; effect size *r*=√(*t* ^2^/[*t* ^2^+df]). Models are adjusted for twins’ length of stay at NICU and mothers’ parity. ^†^ *P*<0.10, *P<0.05, **P<0.01, ***P<0.001.

A significant main effect of gender was found for depression, marital relationship and attitudes to sex. Women had higher depression scores, less positive attitudes to sex, and a more positive perception of their marital relationship than men at baseline.

Marginally significant two-way interactions were found between mode of conception and time for marital relationship, attitudes to pregnancy and the baby and attitudes to sex. Tests of simple slopes revealed that ART parents reported a decline in marital relationship over time (*b*=–0.25, *P*=0.02, *r*=0.36), no changes in attitudes to pregnancy and the baby over time (*P*=0.53), and a slow improvement in attitudes to sex over *postpartum* (*b*=0.65, *P*=0.09, *r*=0.13). In contrast, SC parents reported no changes in marital relationship over time (*P*=0.62), more positive attitudes to pregnancy and the baby over time (*b*=0.27, *P*<0.001, *r*=0.58), and more positive attitudes to sex over *postpartum* (*b*=1.40, *P*<0.001, *r*=0.35). A marginally significant two-way interaction (*P*=0.098) was also found between gender and time for marital relationship indicating that women reported a decline in marital relationship over time (*b*=–0.24, *P*=0.03, *r*=0.34), while men showed no changes in marital relationship over time (*P*=0.51).

**Figure 1:**
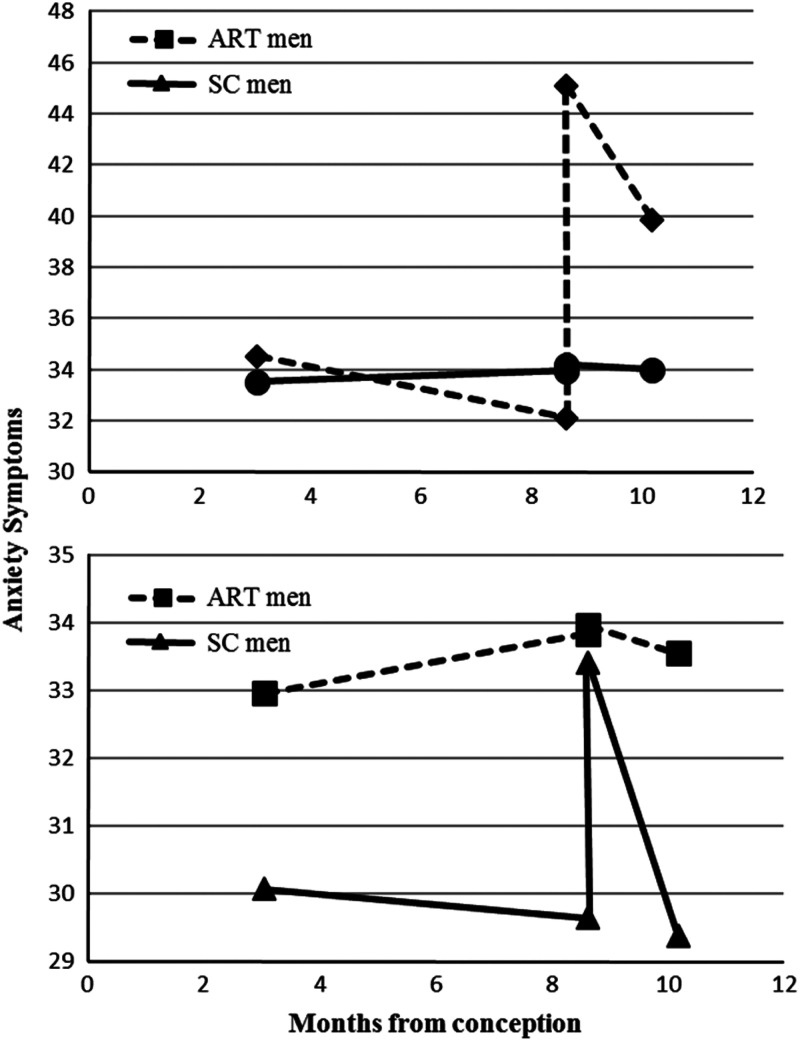
Mean predicted anxiety scores from pregnancy to the *postpartum* period for parents of twins by mode of conception and parents’ gender. ART=assisted reproduction technology; SC=spontaneous conception.

Significant three-way interactions were found between mode of conception, gender and time for anxiety (see Figure 1) and depression. Tests of simple slopes revealed that ART women showed a significant increase in both anxiety (*b*=10.57, *P*=0.01, *r*=0.16), and depression (*b*=3.35, *P*=0.02, *r*=0.16), scores from pregnancy to the *postpartum* period. Smaller but significant increases in anxiety scores (*b*=1.55, *P*=0.04, *r*=0.13) were also noted for SC men from pregnancy to the *postpartum* period. During the *postpartum* period, ART women showed a decline trend in anxiety scores (*b*=−2.99, *P*=0.09, *r*=0.12) and no changes in depression scores (*P*=0.61), whereas a significant decrease in anxiety (*b*=–2.30, *P*=0.02, *r*=0.13) and a decline trend in depression scores (*b*=–0.63, *P*=0.06, *r*=0.12, was observed among SC men. Anxiety and depression scores remained unchanged for ART men and SC women from pregnancy to the *postpartum* period (anxiety: ART men *P*=0.77, SC women *P*=0.79; depression: ART men *P*=0.58, SC women *P*=0.86) and during the *postpartum* period (anxiety: ART men *P*=0.86, SC women *P*=0.93; depression: ART men *P*=0.36, SC women *P*=0.45). Compared with the other groups (SC mothers and fathers, ART fathers), ART women had a higher increase in anxiety (*P*=0.03), and depression scores (*P*=0.07) from pregnancy to the *postpartum* period.

## Discussion

The results of this study show that both mode of conception and parents’ gender are associated with parents’ psychological adjustment across the transition to twin parenthood. Consistent with our hypothesis, ART parents in general and ART mothers in particular showed poor psychological adjustment over the transition to twin parenthood.

ART parents reported a decline in marital relationship quality, no changes in attitudes to pregnancy and the baby and no changes in attitudes to sex over the *postpartum*. In contrast, SC parents did not change their perception of the marital relationship, reported more positive attitudes toward pregnancy and the baby and more positive attitudes to sex over the *postpartum*. These results suggest that ART couples experience interpersonal difficulties during the transition to twin parenthood that could result from several factors, including accumulating stress. Baor and Soskolne (2010) also found that ART mothers of twins report lower marital quality, as well as lower self-efficacy and social support than mothers of SC twins at late pregnancy.

Given that a large proportion of couples undergoing fertility treatments desire having twins (Child *et al*., 2004), it is surprising that ART parents’ attitudes to pregnancy and the baby remained stable, whereas SC parents reported more positive attitudes to pregnancy and the baby over time. However, research with singletons showed that formerly infertile patients tend to experience increased anxiety about the security of the pregnancy and fetal survival (Hammarberg *et al*., 2008). We can speculate that these anxieties are higher in case of ART twin pregnancy, which can ultimately influence the attitudes to pregnancy and the baby. Because the literature on the psychological adjustment in ART and non-ART parents of twins is scarce, the obtained results still need further exploration.

Compared with the other groups (SC mothers and fathers, ART fathers), ART mothers exhibited a significantly higher increase in anxiety and depressive symptoms from pregnancy to the *postpartum* period and only anxiety levels exhibited a decline trend over the *postpartum*. Similar results were obtained even when multiparous SC parents were excluded from the analyses. Our results are consistent with previous studies with mothers of twins showing that ART mothers have lower psychosocial well-being than SC mothers (Colpin *et al*., 1999; Baor *et al*., 2004; Baor and Soskolne, 2010). These adjustment difficulties may be due to a cumulative negative consequence of the prior experience of infertility and fertility treatment (Hammarberg *et al*., 2008). As previously noted, ART parents are a heterogeneous group regarding socio-demographic characteristics (Hammarberg *et al*., 2008), namely because funding policies for fertility treatments vary significantly across countries. In Portugal, ART procedures provided in public clinics/hospitals are fully reimbursed, whereas medications are only partially reimbursed (40%). In the present study, most parents had medium socioeconomic level and medium educational level and no differences in these variables were found between ART and SC groups.

At the couple level, women and men showed similar results at each time period as demonstrated by the correlations at baseline and over time. These results show that couples’ psychological adjustment tends to covary over the transition to twin parenthood. A previous study with singletons has shown that couples’ psychological adjustment is significantly correlated across the transition to parenthood (Tendais and Figueiredo, 2016).

This study brings several clinical and developmental contributions. The longitudinal design starting at early pregnancy and comprising multiple time points covering pregnancy and the early *postpartum* period provided detailed information on changes over time. This information can be helpful for tailoring interventions to the specific needs of parents during the transition to parenthood. In addition, a broad understanding of the psychological adjustment process was gained by the inclusion of measures (marital relationship and pregnancy and child-related attitudes) other than psychological symptoms. Our results also showed that women’s and men’s psychological adjustments are interdependent. Therefore, couple-based interventions (eg, Kalland *et al*., 2016) may be more beneficial for both women and men than those directed at the individual level. These interventions may be helpful for preventing and treating depression and anxiety, especially for ART mothers. Considering that parents’ depressive and anxiety symptoms have been consistently associated with adverse outcomes on the fetus, neonate, and child (Stein *et al*., 2014), self-report measures might be used as a screening measure among parents attending primary care services. Prenatal and postnatal routine care appointments may provide valuable opportunities for early detection and referral for maternal or paternal depression and anxiety, so that psychological support and intervention may begin as early as possible.

The current study has also limitations. First, the small sample size, especially of the ART group, warrants cautious interpretation and generalization of the results. Nevertheless, effect sizes indicate that the identified differences are small to moderate. Second, the SC group included first- and second-time parents, whereas the ART group included only first-time parents, necessitating adjustments for parity. Third, self-report measures may have increased the reporting bias.

Further research is needed to investigate whether the identified early *postpartum* adjustment difficulties are transient or persist over time. In addition, future studies should examine the contribution of declining marital relationship reported by ART couples to the increased anxiety and depressive symptoms during *postpartum* noted in ART women.

In conclusion, the findings of the current study suggest that ART couples may experience greater adjustment difficulties during the transition to twin parenthood than SC couples, especially ART mothers. Although the sample size for the ART group was small, poorer psychological adjustment than the SC group was consistently observed.

## References

[ref1] Baor L , Bar-David J Blickstein I (2004) Psychosocial resource depletion of parents of twins after assisted versus spontaneous reproduction. International Journal of Fertility and Women’s Medicine 49, 13–18. 15038504

[ref2] Baor L Soskolne V (2010) Mothers of IVF and spontaneously conceived twins: a comparison of prenatal maternal expectations, coping resources and maternal stress. Human Reproduction 25, 1490–1496. 20299383 10.1093/humrep/deq045

[ref3] Beck CT (2002) Releasing the pause button: mothering twins during the first year of life. Qualitative Health Research 12, 593–608. 11993558 10.1177/104973202129120124

[ref4] Blondel B , Kogan MD , Alexander GR , Dettani N , Kramer MS Macfarlane A (2002) The impact of the increasing number of multiple births on the rates of preterm birth and low birthweight: an international study. American Journal of Public Health 92, 1323–1330. 12144992 10.2105/ajph.92.8.1323 PMC1447238

[ref5] Child TJ , Henderson AM Tan SL (2004) The desire for multiple pregnancy in male and female infertility patients. Human Reproduction 19, 558–561. 14998951 10.1093/humrep/deh097

[ref6] Choi Y , Bishai D Minkovitz CS (2009) Multiple births are a risk factor for postpartum maternal depressive symptoms. Pediatrics 123, 1147–1154. 19336374 10.1542/peds.2008-1619

[ref7] Colpin H , Munter AD , Nys K Vandemeulebroecke L (1999) Parenting stress and psychosocial well-being among parents with twins conceived naturally or by reproductive technology. Human Reproduction 14, 3133–3137. 10601109 10.1093/humrep/14.12.3133

[ref8] Cox JL , Holden JM Sagovsky R (1987) Detection of postnatal depression: development of the Edinburgh Postnatal Depression Scale. British Journal of Psychiatry 150, 782–786. 10.1192/bjp.150.6.782 3651732

[ref9] Ellison MA Hall JE (2003) Social stigma and compounded losses: quality-of-life issues for multiple-birth families. Fertility and Sterility 80, 405–414. 12909506 10.1016/s0015-0282(03)00659-9

[ref10] Figueiredo B , Mendonça M Sousa R (2004) Versão portuguesa do Maternal Adjustment and Maternal Attitudes (MAMA) [Portuguese version of the maternal adjustment and maternal attitudes (MAMA)]. Psicologia, Saúde & Doenças 5, 31–51.

[ref11] Hammarberg K , Fisher JR Wynter KH (2008) Psychological and social aspects of pregnancy, childbirth and early parenting after assisted conception: a systematic review. Human Reproduction Update 14, 395–414. 18653674 10.1093/humupd/dmn030

[ref12] Hjelmstedt A , Widström A-M , Wramsby H Collins A (2004) Emotional adaptation following successful in vitro fertilization. Fertility and Sterility 81, 1254–1264. 15136086 10.1016/j.fertnstert.2003.09.061

[ref13] Kalland M , Fagerlund Å , von Koskull M Pajulo M (2016) Families First: the development of a new mentalization-based group intervention for first-time parents to promote child development and family health. Primary Health Care Research & Development 17, 3–17. 25827136 10.1017/S146342361500016X PMC4697286

[ref14] Kashy DA , Donnellan MB , Burt SA McGue M (2008) Growth curve models for indistinguishable dyads using multilevel modeling and structural equation modeling: the case of adolescent twins’ conflict with their mothers. Developmental Psychology 44, 316–329. 18331125 10.1037/0012-1649.44.2.316

[ref15] Kenny DA , Kashy DA Cook WL (2006) Dyadic data analysis. New York: The Guilford Press.

[ref16] Kumar R , Robson KM Smith MR (1984) Development of a self-administered questionnaire to measure maternal adjustment and maternal attitudes during pregnancy and after delivery. Journal of Psychosomatic Research 28, 43–51. 6716327 10.1016/0022-3999(84)90039-4

[ref17] Lorenz JM (2012) Neurodevelopmental outcomes of twins. Seminars in Perinatology 36, 201–212. 22713502 10.1053/j.semperi.2012.02.005

[ref18] McMahon CA , Tennant C , Ungerer J Saunders D (1999) ‘Don’t count your chickens’: a comparative study of the experience of pregnancy after IVF conception. Journal of Reproductive and Infant Psychology 17, 345–356.

[ref19] Munro JM , Ironside W Smith GC (1990) Psychiatric morbidity in parents of twins born after in vitro fertilization (IVF) techniques. Journal of In Vitro Fertilization and Embryo Transfer 7, 332–336. 2077086 10.1007/BF01130585

[ref20] Pinto TM , Figueiredo B , Samorinha C , Tendais I Nunes-Costa R (2017) Paternal adjustment and paternal attitudes questionnaire: antenatal and postnatal Portuguese versions. Assessment 24, 820–830. 26658790 10.1177/1073191115621794

[ref21] Pinto TM , Samorinha C , Tendais I , Silva S Figueiredo B (2018) Antenatal paternal adjustment and paternal attitudes after infertility treatment. Human Reproduction 33, 109–115. 29186413 10.1093/humrep/dex349

[ref22] Spielberger CD , Gorsuch RL Lushene RE (1983) The State-Trait Anxiety Inventory: test manual. Palo Alto, CA: Consulting Psychologists Press.

[ref23] Stein A , Pearson RM , Goodman SH , Rapa E , Rahman A , McCallum M , Howard LM Pariante CM (2014) Effects of perinatal mental disorders on the fetus and child. Lancet 15, 1800–1819. 10.1016/S0140-6736(14)61277-0 25455250

[ref24] Tendais I , Costa R , Conde A Figueiredo B (2014) Screening for depression and anxiety disorders from pregnancy to postpartum with the EPDS and STAI. The Spanish Journal of Psychology 17, E7. 25012783 10.1017/sjp.2014.7

[ref25] Tendais I Figueiredo B (2016) Parents’ anxiety and depression symptoms after successful infertility treatment and spontaneous conception: does singleton/twin pregnancy matter? Human Reproduction 31, 2303–2312. 27609986 10.1093/humrep/dew212

[ref26] Vilska S , Unkila-Kallio L , Punamäki RL , Poikkeus P , Repokari L , Sinkkonen J , Tiitinen A Tulppala M (2009) Mental health of mothers and fathers of twins conceived via assisted reproduction treatment: a 1-year prospective study. Human Reproduction 4, 367–377. 10.1093/humrep/den427 19043082

